# Improving Iron and Folic Acid Supplementation Among Pregnant Women: An Implementation Science Approach in East-Central Uganda

**DOI:** 10.9745/GHSP-D-21-00426

**Published:** 2022-12-21

**Authors:** Ahmed K. Luwangula, Laura McGough, Moses Tetui, Henry Wamani, Mark Ssennono, Caroline N. Agabiirwe, Isabelle Michaud-Létourneau, Nathan Tumwesigye, Keith Baleeta, Twaha Rwegyema, Augustin Muhwezi

**Affiliations:** a FHI 360, Kampala, Uganda.; b University Research Co., LLC, Chevy Chase, MD, USA.; c School of Pharmacy, University of Waterloo, Kitchener, Canada; Department of Epidemiology and Global Health, Umeå University, Umeå, Sweden.; d Makerere University, Kampala, Uganda.; e Independent consultant, Kampala, Uganda.; f Society for Improvement Science in Nutrition, Quebec, Canada.; g University Research Co., LLC, Jinja, Uganda.

## Abstract

**Introduction:**

To address maternal iron-deficiency anemia and low uptake of iron and folic acid supplementation (IFAS) among antenatal care (ANC) clinic attendees in East-Central Uganda, the Anemia Implementation Science Initiative embedded enhanced quality improvement (QI) activities into an integrated health project utilizing QI methodologies.

**Methods:**

To address 2 bottlenecks of stock-outs and inadequate health education for pregnant women during ANC, an enhanced QI intervention was implemented from July 2019 to September 2020 in 2 districts. We conducted a mixed-methods effectiveness quasi-experimental study to assess whether the intervention increased the availability of IFAS in the intervention districts. We used longitudinal facility-level data from 2 treatment districts and 1 comparison district for the quantitative results. Difference-in-difference estimation was used to measure the impact of the intervention on IFAS health education and IFA availability at the health facility. We used logistic regression modeling to control for factors associated with IFAS uptake and potential differences in baseline values. Researchers conducted exit interviews with ANC clients and in-depth interviews with providers and district managers for greater insights into the implementation process.

**Results:**

The intervention increased the probability, at a statistically significant level, of pregnant women both receiving IFAS and receiving health education on IFAS during ANC. According to inter-viewees, the intervention approach improved stakeholder engagement and buy-in, which brought about change at all levels of the health system.

**Discussion:**

The intervention successfully addressed the 2 main bottlenecks to availability of IFAS for pregnant women attending ANC—inadequate provision of IFAS education and a weak drug quantification process. Even without additional funds to purchase commodities, this approach improved district capacity to advocate for and manage IFAS commodities. It could also be used to strengthen overall ANC quality.

## Abstract (precis)

An implementation science approach helped successfully identify and address key bottlenecks to the availability of iron and folic acid supplementation for pregnant women attending antenatal care clinics in East-Central Uganda.

## Teaser

**Key Findings:**

- Although iron and folic acid supplementation (IFAS) is a well-known and effective intervention for anemia in pregnant women, its access and uptake were affected by stock-outs and inadequate health education of both health care workers and pregnant women.
- An intervention combined enhanced quality improvement (QI) activities with an implementation science approach that resulted in a significant increase in the probability of pregnant women receiving IFAS and health education on IFAS during antenatal care (ANC).
- The approach improved stakeholder engagement at all levels and helped health workers understand the importance of IFAS, which motivated them to deliver related health education messages and adhere to national IFAS protocols.

**Key Implications:**

- Enhancing QI for IFAS improved and sustained its availability to pregnant women during ANC and transformed health providers into advocates for IFAS.
- Key stakeholder engagement at all levels is critical for objectively identifying the service delivery bottlenecks, gaining consensus, ensuring acceptability, and mobilizing champions for change.

## Introduction

Anemia in pregnancy, defined as having a hemoglobin level of less than 11 g/dl, is a serious public health problem, with the global burden estimated by the World Health Organization to affect approximately 40% of pregnant women. Anemia in pregnancy is associated with several negative peripartum, intrapartum, and postpartum outcomes for the mother, including increased maternal mortality, need for a blood transfusion, and antenatal and postnatal sepsis. In infants, it is associated with impaired cognitive development, increased risk of small for gestation age and low birth weight neonates, and premature births.^1^^–^^5^

Uganda has a supportive policy environment to address the problem of anemia, but major challenges persist. In response to the 2000–2001 Uganda Demographic and Health Survey, which found that 30% of women of reproductive age (15–49 years) were anemic, the Ministry of Health issued the 2002 Uganda National Anemia Policy recommending that all pregnant women are given iron and folic acid supplementation (IFAS) and counseling to prevent and control anemia. IFAS is one of the key interventions aimed at preventing and controlling anemia in women of reproductive age (15–49 years).^6^ This intervention was a major component of the Uganda Nutrition Action Plan (2011–2015)^7^ and was further emphasized in the Uganda Anemia Control and Prevention Strategy (2017–2022).^8^ Despite such a supportive policy environment, anemia prevalence in Uganda remains unacceptably high, with 38% prevalence in pregnant women and 32% in women of reproductive age (nonpregnant).^9^ Four regions in East-Central Uganda, including Busoga (41%), where this intervention was implemented, have a higher prevalence of anemia among pregnant women than the national average.^10^

Although IFAS is a well-known and efficacious intervention to reduce rates of maternal anemia,^11^ implementation challenges often prevent it from fully achieving population-level effectiveness. Specifically, the evidence from efficacy trials does not provide the needed insights on how to overcome the following implementation challenges.

- In Uganda and other East African countries, most women start antenatal care (ANC) halfway through their pregnancy period,^12^^,^^13^ only 30% have their first ANC visit during the first trimester, and many do not complete all the recommended ANC visits, which affects utilization and adherence to IFAS.^14^^–^^15^
- Facilities with poor quality ANC often do not provide anemia-related services in Uganda; quality of ANC is often suboptimal globally and in Uganda specifically, where only 54% of providers adhere to evidence-based guidelines for ANC.^16^^–^^18^
- Across different settings in Africa and Uganda, stock-outs of drugs and supplies have been found to affect the utilization of IFAS. Inadequate supplies are among the major causes of poor adherence to IFAS among pregnant women.^14^^,^^16^

Although IFAS is a well-known and efficacious intervention to reduce rates of anemia during pregnancy, implementation challenges often prevent it from fully achieving population-level effectiveness.

The findings of a bottleneck assessment conducted in 2018 by University Research Co., LLC (URC), the Uganda Ministry of Health (MOH), and partners identified a range of bottlenecks and prioritized 2 of these bottlenecks: (1) inadequate provision of IFAS-related health education to mothers (termed as uncoordinated health education); and (2) a weak drug quantification process at the health facility level resulting in unnecessary stock-outs.

To address these bottlenecks, the Anemia Implementation Science Initiative (Anemia ISI), a 2-year quality improvement (QI) initiative (2018–2020), engaged national and local stakeholders in the design, implementation, and monitoring of a successful intervention to improve availability of IFAS to pregnant women during ANC. We describe how the Anemia ISI was implemented despite the additional implementation challenges imposed by the coronavirus disease (COVID-19) pandemic.

## ANEMIA ISI INTERVENTION

Anemia ISI implementation was embedded in the 2016–2023 Regional Health Integration to Enhance Services in East Central Uganda Activity (RHITES-EC), an integrated project funded by the U.S. Agency for International Development and led by URC that builds district capacity in family planning; maternal, newborn, and child health; HIV; TB; malaria; nutrition; and water, sanitation, and hygiene. To conduct Anemia ISI, URC partnered with the International Initiative for Impact Evaluation and the Society for Implementation Science in Nutrition.

Under the RHITES-EC project, URC supports the Government of Uganda by using the national QI framework, which follows the plan-do-study-act cycle to strengthen health services in all districts where it works. To address the bottlenecks specific to IFAS, however, the project team designed a comprehensive intervention package incorporating enhanced QI support for IFAS with an implementation science approach, especially through the use of the bottleneck assessment tool and stakeholder prioritization workshop. The intervention was implemented at 20 public sector health facilities—10 in each of the 2 implementation arm districts, Buyende and Iganga.

### Processes and Challenges

Implementation took place during an 8-month interval from December 2019 to July 2020, which was interrupted by the COVID-19 pandemic and subsequent restrictions on activities. Because Uganda imposed restrictions on the use of public transport during the lockdown period, many pregnant women were unable to reach facilities for ANC. Health workers also faced challenges in traveling to work. The QI activities that could be conducted virtually—monthly data review meetings and team coaching sessions—continued, but demand for services dropped since many women could not travel to facilities. Lack of personal protective equipment limited the ability of health workers to provide services, especially among community health workers (village health teams). Community health workers often served as key mobilizers for women to attend ANC.

Stakeholder engagement at all levels was critical to the implementation process. In collaboration with the MOH, URC supported the establishment of a national core team to provide strategic and technical guidance to the study. The team included line sectors (MOH and Ministry of Local Government), Makerere University School of Public Health, Office of the Prime Minister, district and facility staff from intervention districts, key implementing partners, and National Medical Stores personnel. The team was engaged through milestone-driven national core team meetings coordinated by URC. The first meeting highlighted stakeholders’ roles in supporting this initiative. Key contributions from the core team included participation in validation of key bottlenecks to guide implementation research and provision of strategic direction based on bottleneck inventory and milestones attained. The Ministry of Local Government provided critically important guidance to overcome bureaucratic redistribution challenges and facilitate interdistrict and interfacility redistributions to alleviate some cases of temporary iron and folic acid (IFA) stock-outs. District-level staff serving as mentors gave on-site support within the intervention arm districts. Key facility support activities included monthly facility performance review meetings by site teams, monthly on-site coaching by district-level mentors, and quarterly collaborative learning sessions by facility teams. These were used to routinely update the bottleneck inventory.

## Methods

We conducted a mixed-methods effectiveness quasi-experimental study to evaluate the intervention’s effect on outcomes using 2 arms: intervention and comparison, analyzed using a difference-in-differences approach.

### Sampling

#### Selection of Health Facilities for Quantitative Data Collection

A total of 30 health facilities (20 health facilities in the intervention arm districts of Buyende and Iganga and 10 in the comparison arm district of Busia) were selected purposively. The districts were selected because they had the highest numbers of ANC attendees for inclusion to offset potential attrition that may occur over time and based on similar district-level population characteristics. Facilities in the district were selected that had a minimum of 100 women attending their first ANC visit during the financial year 2017/2018. The study used cross-sectional facility-level data collected at baseline and endline.

#### Study Arms

In the comparison arm, the QI support focused on addressing gaps in maternal and child health indicators but not specifically on IFAS (Table 1). This was provided by RHITES-EC staff and district health management teams as routine standard of care. In the intervention arm, enhanced support for QI focusing on IFAS was provided through monthly on-site mentorship sessions by both RHITES-EC staff and district health management teams, followed by bimonthly collaborative learning sessions (Table 1).

**TABLE 1.** Study Arms for QI Activities in IFAS Intervention for Pregnant Women Attending ANC in East-Central Uganda

**QI Activities**	**Implementers**	**Study Arms**	
		**Comparison Arm**	**Intervention Arm**
QI performance review and work planning meetings	Health facility QI teams supported by URC/RHITES-EC staff and mentors	Done quarterly Performance indicators guide subsequent mentorship and coaching sessions Not specifically focused on IFAS	Done monthly Subsequent mentorship and coaching done regardless of performance indicators Deeper focus on IFAS Facility data reviews assessed percentage of pregnant women attending ANC and receiving IFAS to monitor performance
Mentorship and coaching sessions	URC/RHITES-EC staff and mentors	Done when needed according to the performance indicators Done by regional mentors	Done monthly Done by district team (better understanding of the environment; longer coaching) District medicine management supervisors conduct monthly on-site mentorships for logistics personnel on stock management specifically focusing on IFAS
Collaborative learning sessions	URC/RHITES-EC staff	Sometimes, not specifically focused on IFAS QI support focused on addressing gaps in maternal and child health indicators but not specifically on IFAS	Done once every 2 months with a focus on harvesting good practices in integrating IFAS messages into health education sessions conducted at ANC; as well as best practices in stock management specific to IFAS

Abbreviations: ANC, antenatal care; IFAS, iron and folic acid supplementation; QI, quality improvement; URC/RHITES-EC, University Research, Co., LLC/Regional Health Integration to Enhance Services in East Central Uganda Activity.

#### Selection of Interviewees for Qualitative Data Collection

At these facilities, researchers conducted ANC exit interviews with each woman (at any point in gestation) who consented to be interviewed until the predetermined sample size for each of the facilities was reached (Table 2).

**TABLE 2.** Distribution of Participants in IFAS Intervention for Pregnant Women Attending ANC in East-Central Uganda

			**Study Participants, No.**	
District	Health Facility Type	Health Facility, No.	**Baseline**	**Endline**
Busia (comparison arm)	HC II	1	10	10
	HC III	7	188	187
	HC IV	1	114	114
	Hospital	1	57	55
Subtotal (comparison arm)		10	369	366
Buyende (intervention arm)	HC II	5	53	53
	HC III	4	78	77
	HC IV	1	25	25
Iganga (intervention arm)	HC II	1	18	7
	HC III	7	99	110
	HC IV	1	16	16
	Hospital	1	79	78
Subtotal (intervention arm)		20	368	366
Total sample size		30	737	732

Abbreviations: ANC, antenatal care; HC, health center; IFAS, iron and folic acid supplementation.

Researchers selected 26 interviewees for key informant interviews based on their involvement in the procurement and dispensing process of IFAS, which included health workers such as medical and clinical officers, midwives, and nurses. In addition, pharmacy technicians, records officers, and store personnel that support the supply chain were also interviewed. Because the qualitative interview guides focused on understanding the changes created through the QI-related intervention being implemented, these interviews were conducted only in the intervention arm. Table 3 presents the categories of key informants interviewed.

**TABLE 3.** Interviewee Categories for Qualitative Interviews in IFAS Intervention for Pregnant Women Attending ANC in East-Central Uganda

**Category**	**Interviewees, No.**
District health team members	3
QI mentors	6
ANC personnel	10
Stores personnel	7
Total	26

Abbreviations: ANC, antenatal care; HC, health center; IFAS, iron and folic acid supplementation; QI, quality improvement.

### Sample Size Calculations

The exit interview respondents’ sample was determined by the difference in means formula (10).

$${n=(Z}_{\infty /2}{\;+\;Z}_{\beta }{)}^{2X}[\pi_{1}(1-\pi_{1})+\pi_{2}(1-\pi_{2})]$$

where n is sample size required in each arm per district; Z_∞/2_ depends on desired significance level of 1.96 (at 95% confidence interval [CI]); Z_β_ depends on desired power of 0.84 (power of 80%); π_1_ is current proportion 0.6 (proportion of women who attend at least 4 or more ANC visits used as a proxy for the study outcomes); and π_2_ is QI support contribution 0.70 (10% increase).

After adjusting for a 10% nonresponse, the total sample size targeted was 370 in each of the study arms. In the comparison arm, 369 were selected at baseline and 366 were selected at endline. In the intervention arm, 368 were selected at baseline and 366 were selected at endline.

### Data Collection

The data collection process was led by URC/RHITES-EC staff and supported by researchers from Makerere University School of Public Health. Baseline data collection occurred from July 23, 2019 to August 9, 2019. Endline data collection occurred August 3–21, 2020. The research team conducted structured exit interviews with pregnant women attending ANC clinics to understand the quality of health education, women’s knowledge and motivation for intake of IFA tablets, and level of satisfaction with availability of IFAS in the health facilities. The semistructured interviews with health workers explored the QI processes, health education for IFAS, and IFAS supply chain processes and tracking procedures. Whereas quantitative data were collected in both arms, qualitative data were only collected in the implementation arm to understand and explore the enhanced QI processes, health education for IFAS, and IFAS supply chain processes and tracking procedures.

### Data Analysis

#### Quantitative Data

We used Open Data Kit for data collection, after which the quantitative data were cleaned and exported into Microsoft Excel and then analyzed using STATA 15. For the main study outcomes, difference-in-differences estimation was used to measure the impact of the intervention on IFAS outcomes. Key factors (sourced from exit interviews with mothers) assessed included maternal age, education, occupation, gestation age, early ANC attendance, and partner support. The main outcomes of interest (receipt of IFAS during ANC, women’s knowledge of the importance of IFAS, and women’s knowledge of the correct number of tablets to take) were selected based on the theory of change (Figure) and expected results of the intervention. Logistic regression modeling was used to control for factors associated with IFAS uptake and potential differences in baseline values. Simple models were used to obtain crude and adjusted odds ratios, corresponding *P* values, and estimates for the difference-in-differences. For secondary outcomes, researchers used descriptive statistics.

**FIGURE:**
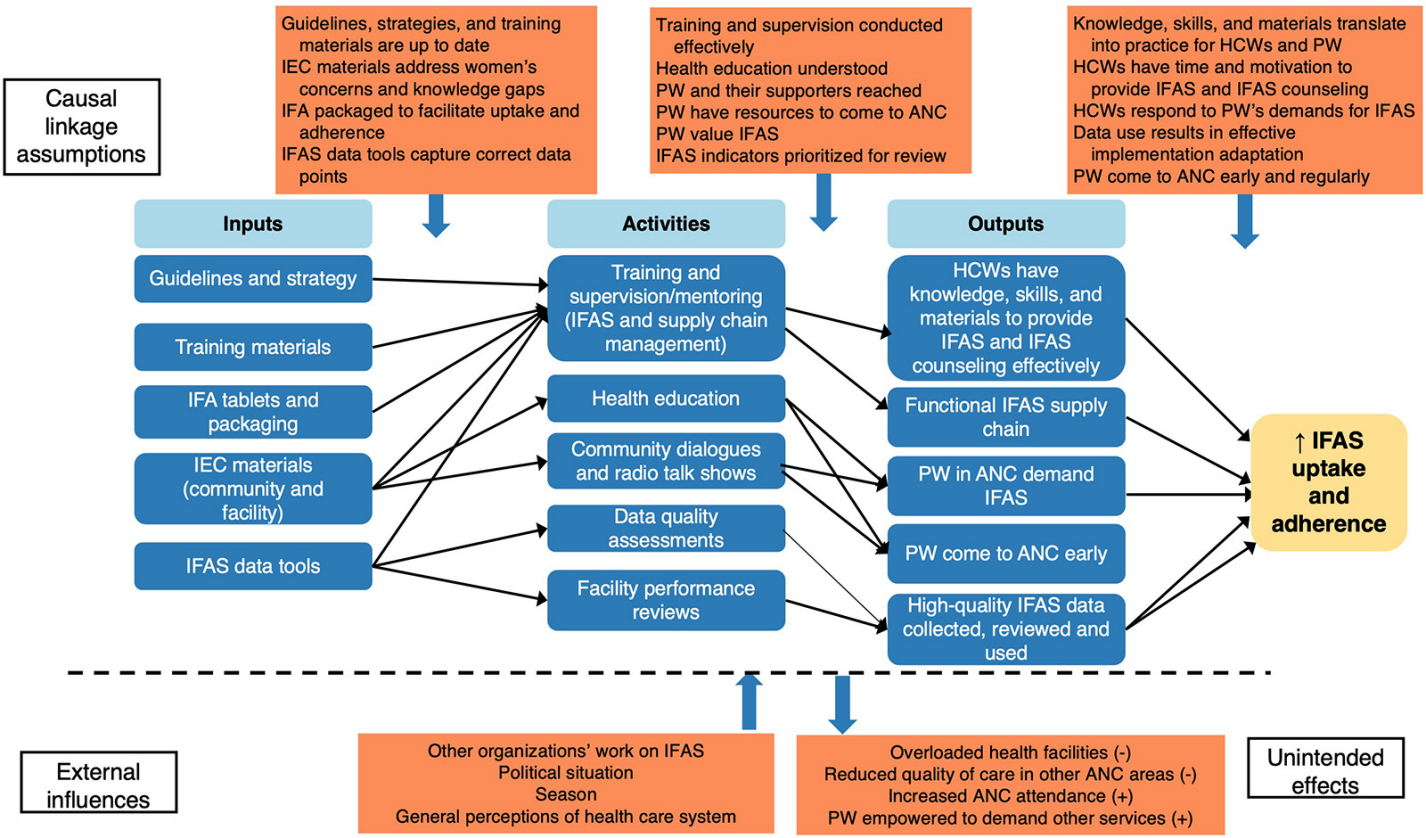
Theory of Change for IFAS Intervention for Pregnant Women in Uganda Abbreviations: ANC, antenatal care; HCW, health care worker; IEC, information, education, and communication; IFA, iron and folic acid; IFAS, iron and folic acid supplementation; PW, pregnant women.

The underlying hypotheses were that improvements in health managers’ and health workers’ ability to manage supply chain processes and deliver quality education about IFAS during ANC, along with women’s improved knowledge of and understanding of the importance of IFAS, would result in increases in the coverage and quantity of IFAS and improve pregnant women’s knowledge/understanding about IFAS. Although the study did not follow up at endline with the same group of pregnant women interviewed at baseline, the 2 groups (baseline and endline) were nonetheless similar in terms of demographics, such as religion, marital status, years of formal education, and primary occupation. Other factors assessed included maternal age, the number of ANC visits, and partner support. The sociodemographic factors for the intervention and comparison samples were comparable for most sociodemographic variables at baseline.

#### Qualitative Data

All transcripts were read several times to ensure familiarity with the data. One of the researchers (MT) led an open coding process supported by the qualitative analysis software MAX QDA for Macs (version 11.2.5). We coded all 26 transcripts, at which point saturation was reached. After the open coding, we undertook a process of grouping and regrouping guided by the objectives of the study.

### Ethical Approval

The study protocol was approved by the TASO ethical review board (TASO REC/015/19-UG-REC-009) and the Uganda National Council of Science and Technology (UNCST-HS396ES). In addition, permission to undertake the study was obtained through the local district authorities in each of the study arms. All the respondents gave their informed written consent, and strict confidentiality was adhered to at the time of data collection, analysis, and sharing of findings.

## RESULTS

### Quantitative Data

#### Sociodemographic Characteristics

The sociodemographic factors for the intervention and comparison samples were comparable for pregnant women’s formal education and marital status but different for religion and occupation (Table 4). Because matching was not done at the individual level, some of the sociodemographic variables may have variances as a result of differences in population composition between comparison and intervention arms; they have not been accounted for in the study design. We address these in the analyses through multivariate logistic regression. Differences in religion in the sample reflect the populations of the respective districts. In the intervention district of Iganga, most of the population is Muslim, while the comparison district is majority Christian.^19^ However, the difference in proportions of religions for pregnant women in the intervention and comparison arms between baseline and endline was not statistically significant (Table 4).

**TABLE 4.** Sociodemographic Characteristics of Sampled Pregnant Women and Key Baseline and Endline Outcomes for IFAS Intervention in East-Central Uganda

	**Baseline**		**Endline**		
	**Intervention, No. (%)**	**Comparison, No. (%)**	**Intervention, No. (%)**	**Comparison, No. (%)**	***P* Value**
Religion					
Anglican	115 (32.9)	121 (32.6)	90 (24.6)	105 (28.8%)	<.001^a^
Pentecostal	45 (12.9)	79 (21.3)	57 (15.6)	104 (28.6%)	
Seventh Day Adventist	3 (0.9)	5 (1.3)	7 (1.9)	2 (0.5)	
Catholic	68 (19.4)	102 (27.5)	76 (20.8)	97 (26.6%)	
Islam	118 (33.7)	59 (15.9)	132 (36.1)	54 (14.8%)	
Other	1 (0.3)	5 (1.3)	4 (1.1)	2 (0.5)	
Primary occupation					
Salaried worker	12 (3.4)	16 (4.4)	26 (7.1)	16 (4.4)	.024^a^
Business owner	55 (15.7)	76 (20.9)	66 (18.0)	76 (20.9)	
Housewife	94 (26.9)	113 (31.0)	92 (25.1)	113 (31.0)	
Peasant farmer	159 (45.4)	133 (36.5)	168 (45.9)	133 (36.5)	
Other	30 (8.6)	26 (7.1)	14 (3.8)	23 (6.3)	
Marital status					
Married	302 (86.3)	290 (78.2)	333 (91.0)	324 (89.0)	.735
Cohabiting	17 (4.9)	46 (12.4)			
Separated	7 (2.0)	7 (1.9)	6 (1.6)	5 (1.4)	
Single	22 (6.3)	27 (7.3)	26 (7.1)	34 (9.3)	
Widowed	2 (0.6)	1 (0.3)	1 (0.3)	1 (0.3)	
Formal education					
Primary	172 (49.1)	161 (43.4)	188 (51.4)	192 (52.7)	.826
Secondary ordinary	78 (22.3)	90 (24.3)	124 (33.9)	119 (32.7)	
Secondary advanced	7 (2.0)	9 (2.4)	6 ( 1.6)	8 (2.2)	
Tertiary	5 (1.4)	6 (1.6)	12 (3.3)	10 (2.7)	
Vocational	4 (1.1)	0 (0.0)	2 (0.5)	5 (1.4)	
None	84 (24.0)	105 (28.3)	34 (9.3)	30 (8.2)	
Gestational trimester at first visit					.124
First trimester	178 (50.9)	189 (50.9)	184 (50.2)	201 (55.2)	
Second trimester	95 (27.1)	133 (35.8)	136 (37.2)	133 (36.5)	
Third trimester	77 (22.0)	49 (13.2)	46 (12.6)	30 (8.3)	

Abbreviation: IFAS, iron and folic acid supplementation.

a *P* value<.05.

#### Pregnant Women’s Receipt of IFAS

The intervention increased the proportion of pregnant women receiving IFAS during ANC (Table 5) with a net intervention effect of 13.1% (i.e., 40.6% in the intervention group versus 27.5% in the comparison group). A statistically significant association was also measured between the quality improvement intervention implemented and women receiving IFAS at the ANC visit, with women in the intervention group having 2 times the odds of receiving IFAS after adjusting for other factors (adjusted OR [aOR]=2.07; 95% CI=1.28, 3.34) (Table 6).

**TABLE 5.** NIE on IFAS Availability for Pregnant Women Receiving ANC in East-Central Uganda

**IFAS Availability**	**Intervention**			**Comparison**			**NIE** ^a^
	**Baseline, No. (%)**	**Endline, No. (%)**	**Estimate of Change, %**	**Baseline, No. (%)**	**Endline, No. (%)**	**Estimate of Change, %**	
Did not receive iron folate tablet	210 (60.0)	71 (19.4)		196 (52.8)	92 (25.3)		
Received iron folate tablet	140 (40.0)	295 (80.6)	40.6	175 (47.2)	272 (74.7)	27.5	13.1

Abbreviations: ANC, antenatal care; IFAS, iron and folic acid supplementation; NIE, net intervention effect.

a The net intervention effect (difference in intervention arm from baseline to endline, minus difference in comparison arm from baseline to endline).

**TABLE 6.** Adjusted Multivariate Model Estimates (Robust) for Receipt of IFAS by Pregnant Women Receiving ANC in East-Central Uganda

**Variables**	**Did not Receive IFAS, No.**	**Received IFAS, No.**	**Crude Odds Ratio** **(95% CI)**	***P* Value**	**Adjusted Odds Ratio** **(95% CI)**	***P* Value**
Treatment dummy						
Control	288	447	1.00		1.00	
Intervention	281	435	0.99 (0.81, 1.23)	.981	0.69 (0.50, 0.94)	.022^a^
Time dummy						
Baseline	406	315	1.00		1.00	
Endline	163	567	4.48 (3.56, 5.61)	<.0001^a^	3.42 (2.46, 4.77)	<.0001^a^
Treatment effect						
0	498	587	1.00		1.00	
1	71	295	3.52 (2.65, 4.68)	<.0001^a^	2.07 (1.28, 3.34)	.003^a^
Age of mother, years						
≤20	178	246	1.00		1.00	
21–30	272	469	1.25 (0.98, 1.59)	.075	1.25 (0.95, 1.63)	.112
≥31	97	139	1.04 (0.75, 1.43)	.826	0.95 (0.65, 1.37)	.802
Education level						
None	108	145	1.00		1.00	
Primary	276	437	1.18 (0.88, 1.57)	.267	0.77 (0.56, 1.08)	.144
Secondary ordinary	163	248	1.13 (0.82, 1.56)	.441	0.67 (0.45, 0.98)	.042^a^
Vocational/Tertiary	10	34	2.53 (1.19, 5.35)	.015^a^	1.87 (0.80, 4.43)	.151
Secondary advanced	12	18	1.12 (0.52, 2.42)	.778	0.97 (0.37, 2.52)	.951
ANC visits						
1	167	268	1.00		1.00	
2	168	223	0.83 (0.63, 1.09)	.181	1.09 (0.75, 1.61)	.638
3	105	198	1.17 (0.87, 1.59)	.301	1.13 (0.80, 1.60)	.491
≥4	129	193	0.93 (0.69, 1.25)	.641	1.23 (0.80, 1.87)	.352
Occupation						
Businessperson/trader	100	152	1.00		1.00	
Housewife	161	268	1.10 (0.80, 1.51)	.577	1.39 (0.96, 2.01)	.085
Peasant farmer	234	371	1.04 (0.77, 1.41)	.783	1.19 (0.83, 1.71)	.349
Salaried worker	29	43	0.98 (0.57, 1.66)	.927	0.82 (0.46, 1.47)	.501
Other	45	48	0.70 (0.43, 1.13)	.147	0.87 (0.51, 1.48)	.598
Gestational trimester at first ANC visit						
First trimester	305	447	1.00		1.00	
Second trimester	176	321	1.24 (0.98, 1.57)	.068	1.33 (0.92, 1.95)	.131
Third trimester	88	114	0.88 (0.65, 1.21)	.441	1.14 (0.77, 1.68)	.490
Partner support						
Did not attend ANC with partner	394	719	1		1	
Attended ANC with partner	175	163	0.51 (0.40, 0.65)	<.0001^a^	0.62 (0.48, 0.81)	<.0001^a^

Abbreviations: ANC, antenatal care; CI, confidence interval; IFAS, iron and folic acid supplementation.

a *P* value<.05.

#### Pregnant Women’s Receipt of Health Education on IFAS

The intervention increased the proportion of pregnant women receiving health education on IFAS during ANC (Table 7) with a net intervention effect of 31.1% (i.e., 18.6% in the intervention group versus −12.5% in the comparison group). Pregnant women in the intervention arm also had a statistically significant increase in the odds of receiving health education on IFAS (aOR=3.5; 95% CI=2.26, 5.42) (Table 8).

**TABLE 7.** NIE on Health Education With IFAS Messaging for Pregnant Women Receiving ANC in East-Central Uganda

**Availability of Health Education on IFAS**	**Intervention**			**Comparison**			**NIE** ^a^
	**Baseline, No. (%)**	**Endline, No. (%)**	**Estimate of Change, %**	**Baseline, No. (%)**	**Endline, No. (%)**	**Estimate of Change, %**	
Didn’t receive health education messages on IFAS	186 (53.3)	127 (34.7)		136 (36.7)	179 (49.2)		
Received health education messages on IFAS	163 (46.7)	239 (65.3)	18.6	235 (63.3)	185 (50.8)	−12.5	31.1

Abbreviations: ANC, antenatal care; IFAS, iron and folic acid supplementation; NIE, net intervention effect.

a The net intervention effect (difference in intervention arm from baseline to endline, minus difference in comparison arm from baseline to endline).

**TABLE 8.** Adjusted Multivariate Model Estimates (Robust) for Receipt of Health Education on IFAS by Pregnant Women Receiving ANC in East-Central Uganda

**Variables**	**Did Not Receive Health Education on IFAS, No.**	**Received Health Education on IFAS, No.**	**Crude Odds Ratio (95% CI)**	***P* Value** ^a^	**Adjusted Odds Ratio (95% CI)**	***P* Value** ^a^
Treatment dummy						
Control	315	420	1.00		1.00	
Intervention	313	402	0.96 (0.78, 1.19)	.724	0.53 (0.38, 0.72)	<.0001
Time dummy						
Baseline	322	398	1.00		1.00	
Endline	306	424	1.12 (0.91, 1.38)	.281	0.59 (0.43, 0.81)	.001
Treatment effect						
0	501	583	1.00		1.00	
1	127	239	1.62 (1.26, 2.07)	<.0001	3.5 (2.26, 5.42)	<.0001
Age of mother, years						
≤20	174	249	1.00		1.00	
21–30	317	424	0.93 (0.73, 1.19)	.585	0.93 (0.73, 1.20)	.593
≥31	105	131	0.87 (0.63, 1.20)	.403	0.94 (0.67, 1.33)	.730
Education level						
None	116	137	1.00		1.00	
Primary	309	403	1.10 (0.83, 1.47)	.500	1.11 (0.81, 1.53)	.497
Secondary	168	243	1.22 (0.89, 1.68)	.209	1.22 (0.85, 1.75)	.291
Vocational/Tertiary	21	23	0.93 (0.49, 1.76)	.818	0.94 (0.46, 1.93)	.875
Secondary advanced level	14	16	0.97 (0.45, 2.06)	.932	0.93 (0.41, 2.07)	.851
ANC visits						
1	186	249	1.00		1.00	
2	179	212	0.88 (0.67, 1.16)	.383	1.02 (0.72, 1.47)	.880
3	134	169	0.94 (0.70, 1.27)	.693	0.94 (0.69, 1.28)	.695
≥4	129	192	1.11 (0.83, 1.49)	.479	1.22 (0.84, 1.81)	.296
Occupation						
Businessperson/trader	119	133	1.00		1.00	
Housewife	186	243	1.16 (0.86, 1.60)	.328	1.14 (0.82, 1.58)	.450
Peasant farmer	254	350	1.23 (0.92, 1.65)	.165	1.20 (0.87, 1.65)	.279
Salaried worker	31	41	1.18 (0.69, 2.01)	.532	1.05 (0.59, 1.89)	.858
Other	38	55	1.30 (0.80, 2.10)	.293	1.35 (0.82, 2.24)	.242
Trimester of first ANC visit						
First trimester	321	430	1		1	
Second trimester	208	289	1.04 (0.83, 1.31)	.755	1.17 (0.84, 1.64)	.343
Third trimester	99	103	0.78 (0.57, 1.06)	.112	0.90 (0.62, 1.29)	.553
Partner support						
Did not attend ANC with partner	499	613	1		1	
Attended ANC with partner	129	209	1.32 (1.02, 1.69)	.030	1.42 (1.09, 1.85)	.010

Abbreviations: ANC, antenatal care; CI, confidence interval; IFAS, iron and folic acid supplementation.

a *P* value<.05.

### Qualitative Data

Researchers selected interviewees based on their involvement in the procurement and dispensing process of IFAS, which included district leaders and health workers such as medical and clinical officers, midwives, and nurses. In addition, pharmacy technicians, records officers, and store personnel that support the supply chain were also interviewed. Because the qualitative interview guides focused on understanding the changes created through the QI-related intervention implemented, these interviews were conducted only in the intervention arm.

## DISCUSSION

The qualitative research results help explain why the combination of enhanced QI activities within an implementation science approach brought about change in a relatively short time amidst the upheavals of the COVID-19 pandemic while also highlighting the challenges of the approach.

The enhanced QI/implementation science approach improved stakeholder engagement and buy-in, which brought about change at all levels of the system, from national to district to facility. Stakeholder buy-in was facilitated through milestone-driven national core team meetings. Following stakeholder acceptance of milestones attained, resolutions were made at each respective meeting to guide implementation. As part of the agenda during the meetings, an update on the ISI bottleneck inventory tool was shared and discussed. Members of the stakeholder meeting provided harmonized strategic direction to guide subsequent activities by the study implementation team. This included (1) guidance on addressing quantification gaps and interdistrict, interfacility stock redistribution, which led to the national care team adopting a simpler and more efficient IFAS facility-to-facility redistribution process; and (2) the prioritization of IFAS as essential in prevention of maternal anemia and related complications, which led to an increase in quantities ordered for and supplied by the National Medical Stores.

A district health team member explains how engaging the National Medical Stores and improving quantification skills at the district level were mutually reinforcing processes that brought about increases in IFA supply at the facility level.

*Before this program came, we were really undergoing a very serious problem of… I can say malnutrition but specifically in this iron and folic line. First of all, we had issues with the delivery system that the amount of folic acid being brought wouldn’t cater for the pregnant women. Yet this is 1 of the districts where we have very many people with malnutrition. When you (Anemia ISI) came in, it helped us a lot in that first of all, we had not actually appreciated the problem. Insofar as the iron and folic was insufficient in terms of supply, it didn’t really hit our minds to think about it so much. Now when it comes to the rounds of making these orders, the DHO calls the in-charges and now they know what to plan for.* —District health team member

Engaging the National Medical Stores and improving quantification skills at the district level were mutually reinforcing processes that brought about increases in IFA supply at the facility level.

In addition, the increase of IFA that was ordered because of an improved understanding of its importance in preventing malnutrition led to increased provider motivation. A provider at an ANC clinic explained how provider behavior changed because of the intervention.

*We now give them thirty tablets for a month. Those days we could get only 5 tins from the store that is 5,000 tablets, so we couldn’t give all the 30 to each mother. That would be the end and we start writing (prescriptions) for them to go and buy (for themselves). But now since we are well stocked, that one (writing prescriptions) we have stopped.* —ANC personnel

Mentorships and embedded learning activities drawing on QI processes also helped providers change their behaviors. Repeat visits by mentors modeled new behaviors and reinforced the importance of IFAS as a critical component of ANC health education. The emphasis on documentation and results—critical components of QI—increased providers’ accountability for IFAS.

*What worked well is that staff were involved, and mentors were ever going to the facilities. So that thing alone helped, and it worked because when staff know that mentors are coming, they can update their journals and they become responsible about the IFAS project.* —District health team member

Equally important, many health workers also noted that having improved their own knowledge of the importance of IFAS made it easier to change their behavior and willingly give pregnant women all the tablets. Previously, rationing was used as a means of ensuring that all pregnant women received some tablets, a practice that the health workers now perceived as negatively affecting pregnant women.

*We had knowledge gaps on issues concerning IFAS because we knew that due to our supply of fewer IFAS that we could economize so that every mother gets, without putting in mind that however much we economize we will be affecting the pregnant women negatively. So, we got to know that every mother is supposed to take IFAS almost throughout pregnancy, we were also just reluctant at counting all 30 tablets, but now we know its importance.* —QI mentor

Exit interviews also showed increases in client satisfaction and willingness to take IFAS. Over 75% of the pregnant women were conversant with the importance of taking IFAS both at baseline and endline and in both study arms. The proportion of pregnant women who acknowledged that through health education they learned the importance of taking IFAS increased from 6.9% to 35.2% in the intervention arm; and from 9.4% to 29.6% in comparison between baseline and endline, respectively.

The proportion of pregnant women who acknowledged that through health education they learned the importance of taking IFAS increased from 6.9% to 35.2% in the intervention arm.

One challenge of QI approaches is the amount of time required from already busy facility staff who must juggle the QI activities alongside their daily routines, especially in facilities with weak or nonfunctional QI teams.

*The disadvantage, at times, you find people are busy, so you get little time to share with the colleagues at times because you can find them, they are very busy. Again, you have to sit, either you wait until they finish, or they will not concentrate.* —QI mentor

Part of the challenge was that some health workers were not familiar with QI processes and systems, which led to extra time spent on reinforcing QI concepts and processes.

Finally, although an enhanced QI approach provides a systematic way of understanding barriers, it is important to note that such an approach cannot address all structural and health system barriers. Examples of barriers we noted include (1) a lack of standardized guidelines for health education during ANC and (2) a perception by health care workers in ANC clinics that packaging IFA tablets in tins creates too much additional work.

### Limitations

We observed that utilizing an enhanced QI approach was associated with a significantly increased probability of pregnant women receiving IFAS during ANC. However, because this study was embedded in an ongoing project with preplanned activities, randomization of study districts and health facilities was not an option. The lack of randomization limits the ability of this study to draw conclusions on a temporal or causal association between an enhanced QI approach and the increased probability of pregnant women receiving IFAS during ANC.

We know that health professionals talk and share among themselves in both formal (e.g., mentorships or quality improvement collaborative meetings) and informal settings within and outside of their districts. Thus, it is possible that health facilities in the control district (Busia) could have adopted best practices from implementation districts (Buyende and Iganga). This potential for contamination was reduced by the selection of implementation and control districts that do not share physical borders.

In the data analysis, a multivariate logistic regression was performed. Analysis was limited to determining the treatment effect of the intervention and, accordingly, the multivariate logistic regression to address biases between the control and intervention groups, which may not have addressed all unmeasured and unknown confounders.

## Lessons Learned

The Anemia ISI undertook a comprehensive analysis of implementation through the identification and timely engagement of key stakeholders at all levels, a bottleneck assessment, and the validation of bottlenecks to be adapted to contextual needs. Specific lessons learned during implementation include the following.

Key stakeholder engagement at all levels (including MOH at district and facility levels and civil society) was critical for objectively identifying the bottlenecks to IFAS, gaining consensus, ensuring acceptability, and preparing change champions for the enhanced QI interventions to address IFAS bottlenecks at the health facility, district, regional, and national levels. This level of engagement enables acceptability, ownership, and institutionalization of best practices and approaches to address performance gaps.

The national core meetings provided a platform for advocacy on the sustainability of best practices in IFAS to key national players, including the MOH and the National Medical Stores; on the importance of IFAS for maternal health; and to build further on commitments to ensure consistent supply of IFA tablets through resource allocation, accurate quantification, and timely delivery to health facilities.

Enhanced QI approaches are effective strategies for addressing process gaps caused by lack of health education and stock management of IFA. Implementing the approaches with fidelity can significantly improve availability of IFA tablets for mothers and IFAS uptake among pregnant mothers attending ANC.

Engaging health workers through on-site mentorships changed health care provider behavior regarding quantification and ordering of IFA, which resulted in increased availability of IFA in facilities where enhanced QI interventions were implemented.

The success of the stakeholder engagement and enhanced QI interventions in significantly improving uptake of IFAS, as well as addressing underlying barriers and improving acceptability among health care providers, gives an opportunity to adapt and leverage these same approaches to improve other aspects of ANC care. These may include addressing barriers to timely ANC attendance, preventing malaria in pregnancy through intermittent preventive treatment in pregnancy, and improving health facility delivery rates for pregnant mothers.

Despite the benefits of the enhanced QI approaches in improving the availability and uptake of IFAS, several health care providers noted that the intervention is time consuming. Because of the extra time required to improve staff understanding of QI processes, this intervention is most likely to succeed in places where functioning QI systems and teams already exist.

Due to the extra time required to improve staff understanding of QI processes, this intervention is most likely to succeed in places where functioning QI systems and teams already exist.

Beyond service delivery and access to essential medicines, the approach can further be used to identify and address bottlenecks in other components of the health system (e.g., workforce, information systems, financing, and leadership) and at all system levels (from facility to national level).

## CONCLUSION

This study provides evidence that an enhanced QI intervention can increase the odds of a pregnant woman receiving both IFA and health education with IFAS messages during ANC at a statistically significant level. By identifying and prioritizing bottlenecks to the successful uptake of IFAS, district- and facility-level QI teams were able to focus QI interventions on improving the weak IFAS quantification process and poor quality of health education on IFAS during ANC. This approach is best suited to health systems with functioning QI teams and has the potential to improve the overall quality of ANC.

## Notes

**Peer Reviewed**

**First published online:** December 12, 2022.
